# Matteucinol combined with temozolomide inhibits glioblastoma proliferation, invasion, and progression: an *in vitro*, *in silico*, and *in vivo* study

**DOI:** 10.1590/1414-431X2022e12076

**Published:** 2022-08-22

**Authors:** J.B. Netto, E.S.A. Melo, A.G.S. Oliveira, L.R. Sousa, L.R. Santiago, D.M. Santos, R.C.R. Chagas, A.S. Gonçalves, R.G. Thomé, H.B. Santos, R.M. Reis, R.I.M.A. Ribeiro

**Affiliations:** 1 Laboratório de Patologia Experimental, Universidade Federal de São João del-Rei, Divinópolis, MG, Brasil; 2 UNA, Unidade Guajajaras, Belo Horizonte, MG, Brasil; 3 Laboratório de Processamento de Tecidos, Universidade Federal de São João del-Rei, Divinópolis, MG, Brasil; 4 Instituto Federal de Educação, Ciência e Tecnologia do Espírito Santo, Vila Velha, ES, Brasil; 5 Universidade Federal do Espírito Santo, Goiabeiras, ES, Brasil; 6 Centro de Pesquisa em Oncologia Molecular, Hospital do Câncer de Barretos, Barretos, SP, Brasil; 7 Life and Health Sciences Research Institute (ICVS), School of Medicine, University of Minho, Braga, Portugal; 8 ICVS/3B's - PT Government Associate Laboratory, Braga/Guimarães, Portugal

**Keywords:** Brain cancer, Antitumor, Natural products, Apoptosis

## Abstract

Glioblastoma is the most prevalent and malignant brain tumor identified in adults. Surgical resection followed by radiotherapy and chemotherapy, mainly with temozolomide (TMZ), is the chosen treatment for this type of tumor. However, the average survival of patients is around 15 months. Novel approaches to glioblastoma treatment are greatly needed. Here, we aimed to investigate the anti-glioblastoma effect of the combination of matteucinol (Mat) (dihydroxyflavanone derived from *Miconia chamissois* Naudin) with the chemotherapeutic TMZ *in vitro* using tumor (U-251MG) and normal astrocyte (NHA) cell lines and *in vivo* using the chick embryo chorioallantoic membrane (CAM) assay. The combination was cytotoxic and selective for tumor cells (28 μg/mL Mat and 9.71 μg/mL TMZ). Additionally, the combination did not alter cell adhesion but caused morphological changes characteristic of apoptosis *in vitro*. Notably, the combination was also able to reduce tumor growth in the chick embryo model (CAM assay). The docking results showed that Mat was the best ligand to the cell death membrane receptor TNFR1 and to TNFR1/TMZ complex, suggesting that these two molecules may be working together increasing their potential. In conclusion, Mat-TMZ can be a good candidate for pharmacokinetic studies in view of clinical use for the treatment of glioblastoma.

## Introduction

Glioblastoma is the most prevalent malignant brain tumor in adults. It is characterized by marked aggressiveness and notoriously invasive behavior, leading to its classification as a grade 4 central nervous system tumor, according to the World Health Organization (WHO) (1). Glioblastoma has an average incidence of 3 cases per 100,000 individuals, corresponding to approximately 60% of existing malignant gliomas (2). The unique anatomy of the brain poses a high risk of injury to areas adjacent to the tumor, which leads to a poor prognosis for this disease, as it makes the extensive cytoreduction impossible, making the degree of resections extremely variable, according to the specifics of each patient (1). In fact, neurosurgery alone is not sufficient as a therapeutic intervention for this type of malignant glioma and warrants adjuvant treatment with radiotherapy and/or chemotherapy.

Alongside several chemotherapeutic drugs approved up to 2005, the orally-administered alkylating agent temozolomide (TMZ) has been the primary advance to date (3), with a high level of penetration of the blood-brain barrier (BBB) (96-100%) due to its low molecular weight (194.154 g/mol). After oral administration, the prodrug TMZ is quickly absorbed in the small intestine and then undergoes spontaneous intracellular conversion into a potent methylating agent, 5-(3-methyl-1-triazeno)imidazole-4-carboxamide (MTIC). MTIC methylates DNA at the N7 and O6 positions of guanine residues. Methylation of the base leads to inefficient repair of these breaks, resulting in apoptosis (4). Unfortunately, the effect of TMZ is usually only palliative (3).

Identification of new chemotherapeutic treatments alone or in combination thus became the aim of several studies involving glioblastoma. Unpublished data from our research group demonstrated that extracts of *Miconia chamissois* Naudin inhibited the activity of matrix metalloproteinases (MMP-2 and -9) (Alves NR et al., unpublished results). We also identified a flavanone, Matteucinol (Mat), in this species (5). Previously, Mat also was identified in two other species from the same genus, *Miconia trailii* and *Miconia prasina* (2), and *Rhododendron hainanense* (6). In addition, flavonoids can cross the BBB through transcellular diffusion, carrier-mediated transcellular transport, or paracellular diffusion (7).

Given that the antitumor activity of the Mat and TMZ combination has great potential for the treatment of glioblastoma (5), and that its mechanism of action is still not elucidated, we speculate that the effect of Mat-TMZ (28 μg/mL of matteucinol and 9.7 μg/mL of temozolomide) used by Silva et al. (5) was selective and can act in important processes inherent to cell invasion and metastasis. In the present study, we investigated the biological action of Mat-TMZ using U-251MG human glioblastoma cells as an *in vitro* model and an *in vivo* chicken embryo chorioallantoic membrane (CAM) assay.

## Material and Methods

### Plant material and extraction

*Miconia chamissois* leaves were collected in June 2015 from the Brazilian Cerrado (18°54'5066''S and 48°13'5728''W). A sample of the plant was identified and deposited in the herbarium of the Federal University of Uberlândia (HUFU59592). The leaves were ground into powder and subjected to extraction in an aqueous hydroethanolic solution (7:3) for 72 h at room temperature and then filtered. The extract was freeze-dried (Liotop K105, Liobras, Brazil) to obtain a crude extract (CE). The chloroform partition (CP) was obtained by liquid-liquid extraction. The CP was fractionated over a silica gel column 60G (0.05-0.20 mm 170-270 mesh) and eluted with chloroform and chloroform/ethyl acetate (20:80 v/v; 50:50 v/v; and 80:20 v/v). After analyzing the chromatographic profile, the obtained fractions (McC1) were pooled and freeze-dried. Matteucinol (8 mg) was isolated by silica gel flash column chromatography. The sample was eluted with chloroform/ethyl acetate (30:1; v/v) and ethyl acetate (5).

### Cell viability assay

Human glioblastoma U-251 MG cells and normal human astrocytes (NHA) cell line were obtained from the European Collection of Cell Cultures (Salisbury, UK). Cells were seeded in 96-well plates with 5×10^3^ cells per well and then incubated overnight in a CO_2_ incubator (5%) at 37°C for adherence to the plate. The cell lines were grown in DMEM (Dulbecco's modified eagle medium) with 1% P/S antibiotics (penicillin/streptomycin) and supplemented with 10% FBS (fetal bovine serum). Treatment was prepared in dimethyl sulfoxide (DMSO) (1%) and DMEM (0.5% FBS). The cells were treated with Mat-TMZ (CAS No. 85622-93-1, ≥99%, Tocris Bioscience, UK), 28 μg/mL Mat, and 9.7 μg/mL TMZ for 72 h. DMSO (1%) was used for negative control. Cell viability was quantified using the colorimetric reagent MTT (3-[4,5-dimethylthiazol-2-yl]-2,5-diphenyltetrazolium bromide; M6494, Invitrogen, USA). When in contact with viable cells, this compound is reduced to formazan by mitochondrial dehydrogenases. The metabolism of the compound is directly proportional to the number of viable cells. Absorbance was measured using an ELISA plate reader at a wavelength of 570 nm after 3 h of incubation with 10 μL of MTT (0.5 mg/mL) diluted in 90 μL of DMEM with 0.5% FBS, the medium was removed from the 96‐well plate, and 100 μL DMSO (99.9%; Synth, Brazil) was added to each well. Absorbance measurements were converted into mean percentage ± standard deviation (SD) of cell viability and normalized against the control. Thus, the results are reported as percent cell viability. All experiments were performed with cells in the third or fourth passes, in triplicate, and in two independent experiments.

### Cell adhesion

Cell adhesion assay was performed as previously described with some modifications (8). The wells of a 96-well plate were covered with 100 μL of Matrigel™ diluted in bovine serum albumin (BSA) (10 μg/mL) (1:10; Corning, USA, REF. 354234) and then incubated for 24 h in a CO_2_ incubator at 37°C. Excess liquid was removed, and 100 μL of BSA solution (0.1%) was added to the plate before incubation for 2 h under the same conditions. The solution was removed, and the wells were washed with phosphate buffered saline (PBS), followed by feeding the U-251 MG cells (6×10^3^ cells/well) with DMEM (without FBS) containing the treatment. The cells were incubated for another 2 h in a CO_2_ incubator at 37°C. The cells were fixed with 10% trichloroacetic acid (TCA) for 1 h and stained with a crystal violet solution. Following this, 100 μL of 5 mM Tris was added to the wells, which were then read using a spectrophotometer (Biotek, USA) at a wavelength of 540 nm. The results of the absorbance were converted into a mean percentage ± SD and normalized against the control.

### Apoptosis assay

U-251 MG cells resuspended in DMEM with 10% FBS were seeded in a 96-well plate at a density of 75×10^3^ cells/well and incubated for 24 h in a CO_2_ incubator. The medium was removed, and Mat-TMZ was added with DMEM (0.5% FBS). The plate was then incubated for 24 h. Following this, the medium was removed, and the cells were washed with 100 μL of PBS. Then, 10 μL of propidium iodide (PI) (0.01 mg/mL) and 10 μL of acridine orange (AO) (0.01 mg/mL) were added per well and gently homogenized. Twenty images per well were obtained at 200× magnification using the Axio Vert.A1 microscope (Germany). Morphological analysis was carried out according to Rahman et al. (9). Finally, the cells were quantified using the Zen software (Zeiss, Germany), and the data were converted to mean percentage ± SD and normalized against the control.

### Molecular docking study

**Figure 1:**
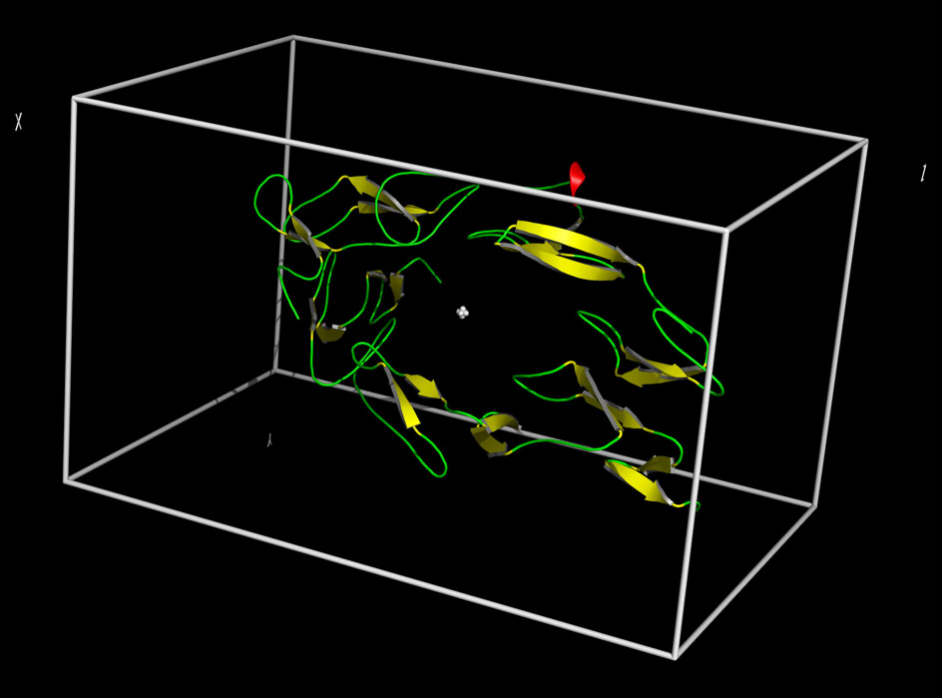
3D structure of TNFR1 with gridbox used for docking simulations. TNFR: tumor necrosis factor receptor.

The three-dimensional structure of tumor necrosis factor receptor 1 (TNFR1) was retrieved from the protein data bank (PDB, ID: 1NCF) archive. The three-dimensional models were verified and repaired using the WHAT IF server (10). The structures of Mat and TMZ were retrieved from PubChem (https://pubchem.ncbi.nlm.nih.gov/) and submitted to 3D optimization using the Ghemical software (11) and TRIPOS 5.2 force field (12). Thereafter, the .pdb files of the ligands and the structure of TNFR1 were opened using the ADT program (13) for the preparation of the input files for docking analysis. TNFR1 and ligands were treated with united atom approximation by merging the nonpolar hydrogen atoms. The torsion angles, defined by the ADT program (13), were used to account for flexibility in the compounds. A three-dimensional grid was created in ADT to evaluate the binding energy between ligands and protein. The grid maps were separated by 0.1 nm and defined using a simple text file (conf.txt) with the following flags: receptor = TNFR1.pdbqt; center_x = 21.259; center_y = 14.649; center_z = 35.553; size_x = 56; size_y = 54; size_z = 90; cpu = 16; and num_modes = 20. This amounted to a total of 272160 Å3 (Figure 1).

The docking calculations were carried out using the AutoDock Vina program (14). Twenty docking rounds were performed for each system [system 1: TNFR1, system 2: TNFR1/TMZ, and system 3: TNFR1/Mat], docking of Mat at system 1, TMZ at system 1; Mat at system 2 and TMZ at system 3. The choice of the best docked ligand was based on the lowest binding energy.

### Chick embryo chorioallantoic membrane (CAM) assay

For the CAM assay, fertilized eggs from the Rivelli hatchery (Mateus Leme, Minas Gerais, Brazil) were obtained. The sanitized eggs were kept horizontally in a digital automatic brooder under controlled conditions of humidity (60%) and temperature (37°C). Two holes were made on both sides of the eggs on the third day of incubation, one in the region of the air chamber and the other at the opposite end, allowing the removal of the chorioallantoic membrane from the upper region of the eggshell to open a 15 mm diameter “window” with sterile tweezers and scissors. On the tenth day of incubation, 2×10^6^ cells (U-251 MG) were resuspended in 20 μL of Matrigel™ and applied to the chorioallantoic membrane. On the 14th day, the tumors on the membranes were photographed *in ovo*, with a MOTIC stereomicroscope (Moticam 580 5.0 MP, Hong Kong) using the 0.5× objective. The Mat-TMZ treatment was applied in a single dose, and the eggs (n=10) were covered with adhesive tape and incubated again. The control group (n=10) was injected with an equal volume of Matrigel™. Tumors and membranes were photographed again *in ovo* 72 h after treatment (5). Finally, embryos were euthanized in the freezer at -80°C for 10 min, according to the parameters described by the AVMA guidelines (15). Subsequently, CAMs with the tumors were removed and fixed with 4% paraformaldehyde for 20 s. The fixed membranes were placed on a plate with FBS and photographed using the stereomicroscope at 1× magnification. The blood vessels in the *ex ovo* images of the membranes were quantified using the AngioTool software (UK), where the percentage of the area consisting of blood vessels and the number of vessel junctions were evaluated. The *in ovo* images, taken on the 14th day and on the 17th day, were used to measure the tumors' area in square pixels, and the results are reported as mean percentage of tumor growth ± SD (16). All CAMs were used in all analyses. This assay was approved by the Animal Research Ethics Committee of the Federal University of São João del-Rei (approval number 039/2017). The selectivity assay was performed in experimental triplicates and biological duplicates (two independent assays). The rest of the *in vitro* tests were carried out in experimental and biological duplicates. The *in vivo* experiment used 10 animals (CAM) in each group (controls and treated).

### Statistical analyses

The results were analyzed using the GraphPad Prism software (version 5.01, USA). The level of significance used in all statistical analyses was P<0.05. Data are reported as means±SD. Student's *t*-test was used to compare means between groups.

## Results

### Selectivity of Mat-TMZ through tumor cell line

**Figure 2:**
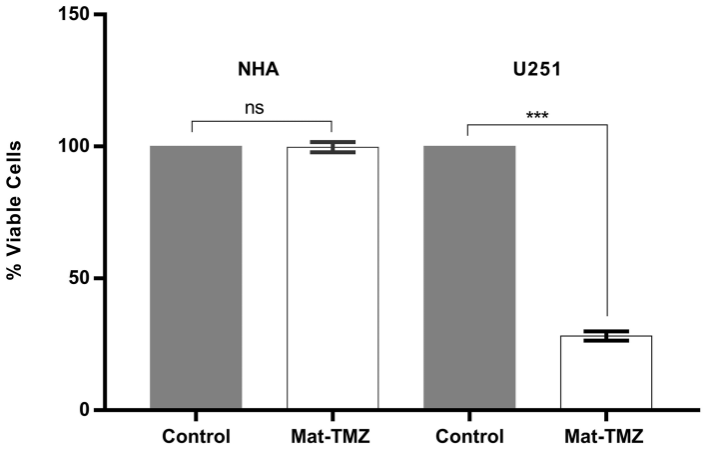
Tumor (U251) and normal (NHA) cell viability after 72 h of treatment with matteucinol-temozolomide (Mat-TMZ) (28 μg/mL Mat and 9.71 μg/mL TMZ). The assay was performed in experimental triplicates and two independent experiments. The negative control used was 1% DMSO. Data are reported as means±SD. ***P=0.0003 (Student's *t*-test). ns: not significant.

Treatment with Mat-TMZ (28 μg/mL Mat and 9.71 μg/ml TMZ) did not cause a significant reduction in cell viability in the normal astrocyte cell line (NHA). Therefore, this combination selectively reduced cell viability in the tumor cell line (U-251 MG) (Figure 2).

### Cell adhesion after treatment with MAT-TMZ

There was no significant reduction in cell adhesion after a 72-h treatment with Mat-TMZ at its IC_50_ (28 μg/mL Mat and 9.71 μg/mL TMZ) .

### Apoptosis induction

**Figure 3:**
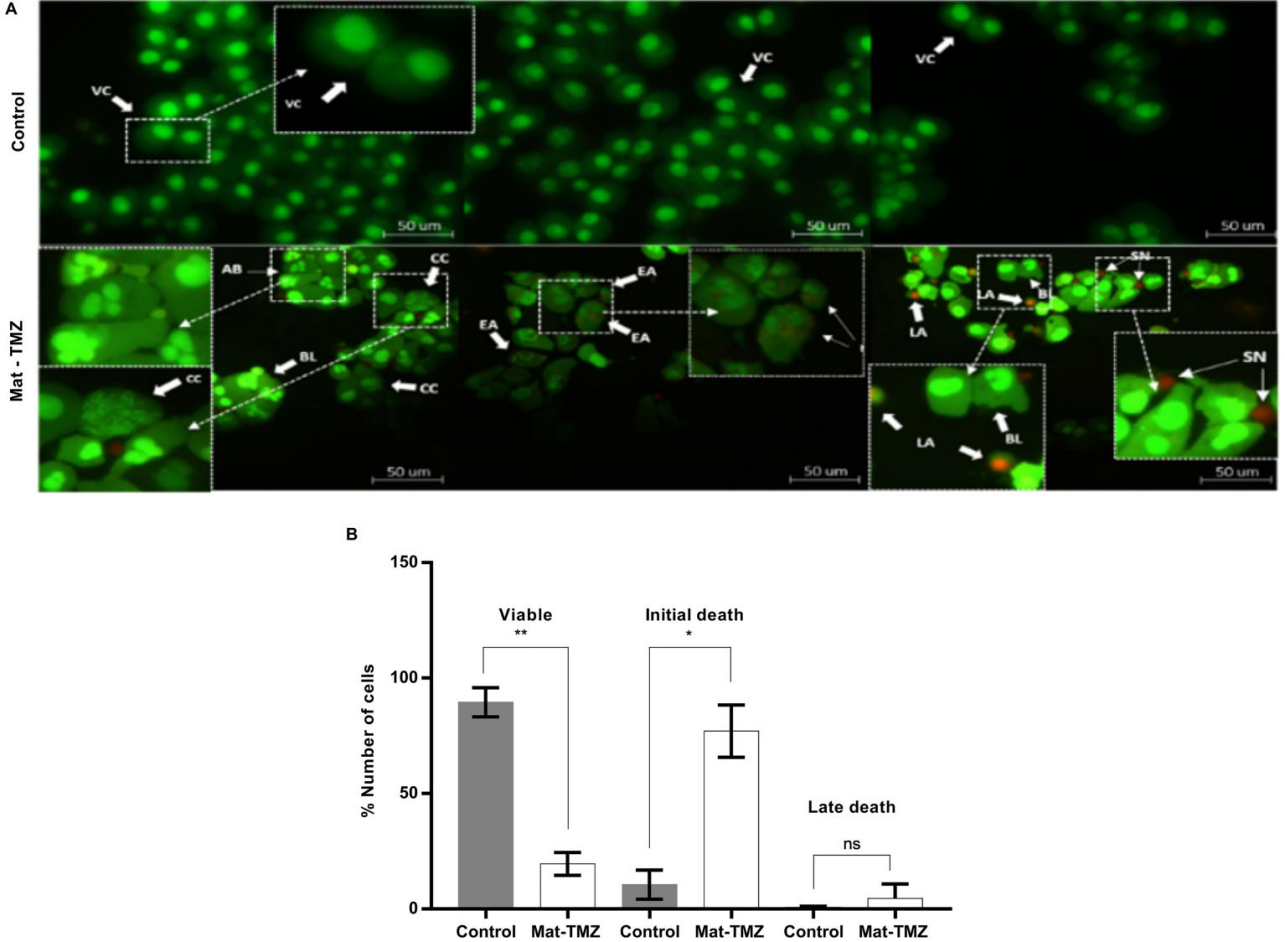
**A**, Fluorescence morphological assay using acridine orange and propidium iodide in U251 cell line after treatment with matteucinol-temozolomide (Mat-TMZ) (28 μg/mL Mat and 9.71 μg/mL TMZ). The assay was performed in experimental duplicates and two independent experiments. The negative control was 1% DMSO. **B**, Number of viable and dead cells in the U251 cell line. Data are reported as means±SD. *P=0.0185; **P=0.0066 (Student's *t*-test). ns: not significant. VC: viable cells; EA: early apoptotic cells; CC: chromatin condensation; BL: cell membrane blubbing; AB: apoptotic body; LA: late apoptotic cells; SN: secondary necrotic cell.

The morphology of the tumor cell line U-251 MG was assessed after treatment with Mat-TMZ and double staining with AO and PI. Condensation and fragmentation of nuclei (FN) and cell membrane blebbing (BL) were observed, and a large number of cells were marked in red by PI (Figure 3). After 24 h of treatment with the combination, we observed a significant reduction in cell viability (P=0.0066), showing only 19% viable cells. In addition, there was a significant increase in the number of early cell death (P=0.0185), representing 77%. About 4% of the cells were in late death after treatment. These characteristics (nucleus condensation, cell membrane blebbing, and cell fragmentation) are typical of apoptosis.

### Molecular docking study

**Figure 4:**
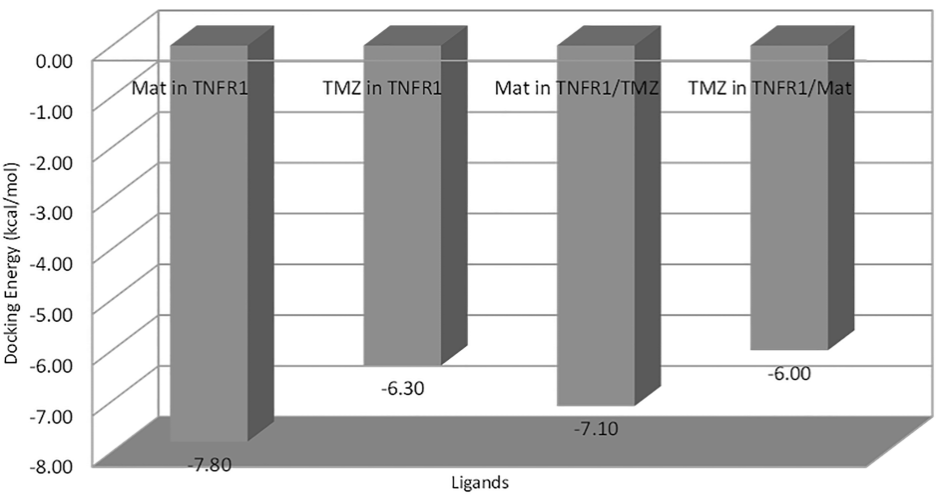
Docking energy for each protein/ligand complex. Mat: matteucinol; TMZ: temozolomide.

Energy docking results showed that Mat was a better ligand than TMZ, concerning the TNFR1 receptor alone and the TNFR1/TMZ complex (Figure 4, Supplementary Figure S1).

**Figure 5 f05:**
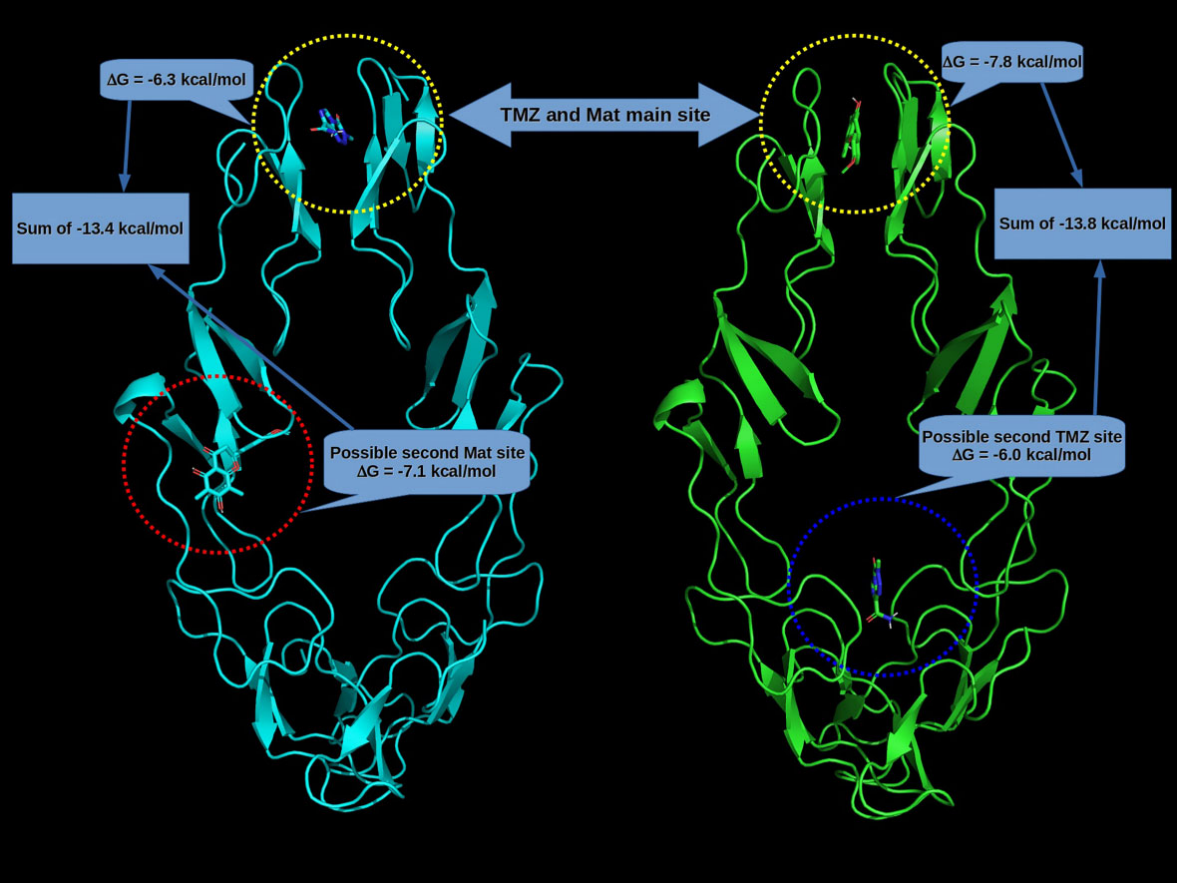
Two binding sites between TNFR1 (tumor necrosis factor receptor), matteucinol (Mat), and temozolomide (TMZ) with their docking energy.

Docking results showed that Mat and TMZ were docked at the same site in the TNFR1 and at two different sites when docked, respectively, in TNFR1/TMZ and TNFR1/Matt complexes suggesting that these two molecules may be acting together increasing their potential (Figure 5).

**Figure 6:**
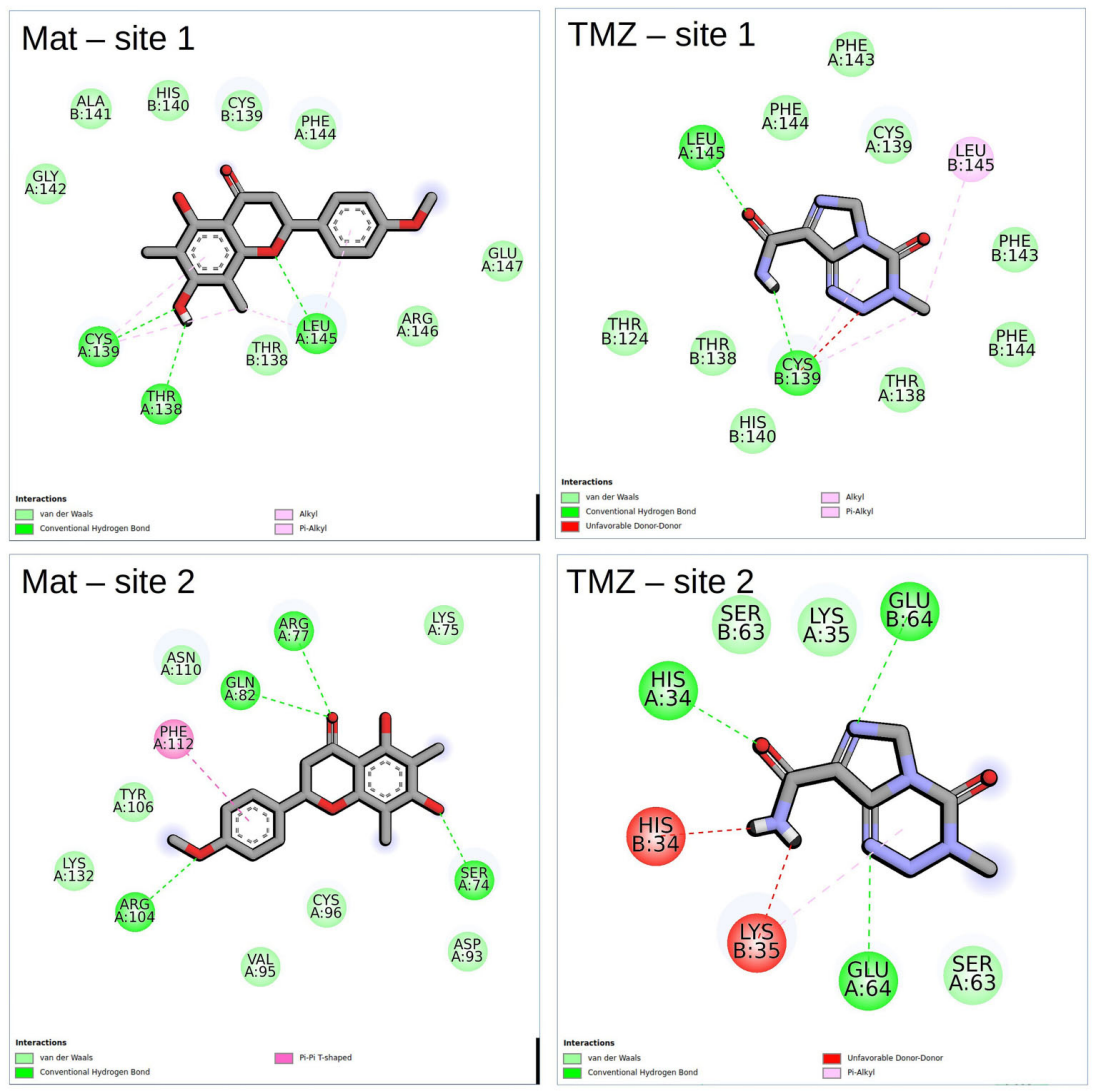
2D interaction map of matteucinol (Mat), temozolomide (TMZ), and tumor necrosis factor receptor (TNFR1).

To study the type of interactions that keep the ligands into the two binding sites, the 2D map interaction between the ligands and TNFR1 was carried out using the Discovery Studio Visualizer software (https://discover.3ds.com/discovery-studio-visualizer-download) (Figure 6). The 2D visualization map of docking results showed that in relation to site 1, Mat interacts with 10 amino acids, making 3 hydrogen bonds, respectively with Cys139, Leu145, and Thr138 and TMZ with 7 amino acids, making 2 hydrogen bonds, respectively, with Leu145 and Cys139, suggesting that Leu145 and Cys139 may be important inhibition residues. In relation to possible interaction site 2, Mat interacted with 12 amino acids making 4 hydrogen bonds, respectively, with Arg104, Gln82, Arg77, and Ser74 while TMZ interacted in another region and with 5 amino acids, being His34 and Glu34, respectively, by one and two hydrogen bonds totalizing 3 polar contacts (Figures 4-6).

### Tumor growth inhibition after Mat-TMZ treatment *in vivo*

**Figure 7:**
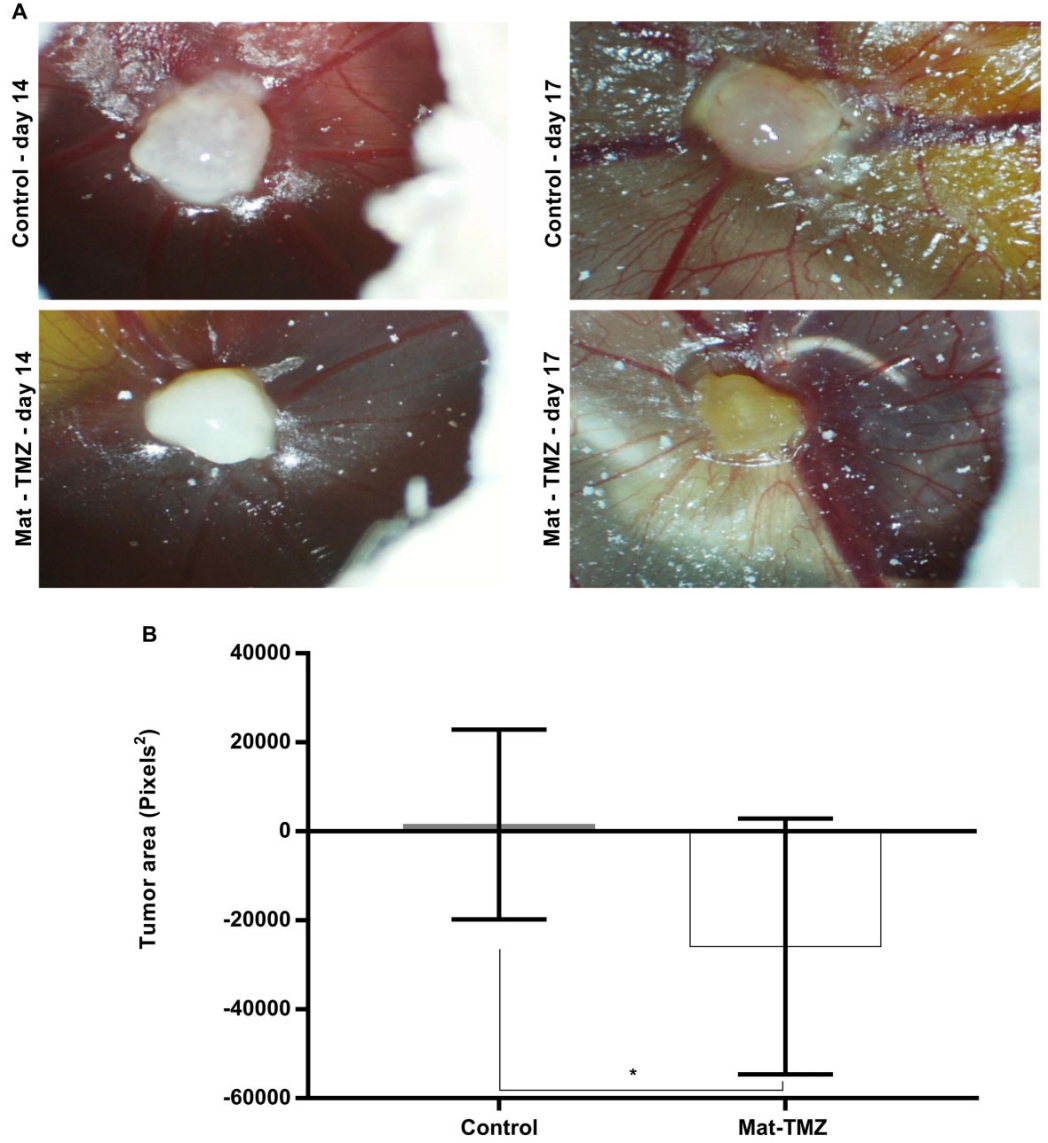
Change in tumor area *in vivo* in the chorioallantoic membrane assay after treatment with matteucinol-temozolomide (Mat-TMZ). **A**, Representative images of the control and treated group on the 14th and 17th days. **B**, Tumor area was measured in square pixels. Data are reported as mean percent tumor growth ± SD. *P=0.0238 (Student's *t*-test).

Tumor growth of the U-251 MG cell line was evaluated *in vivo* using the CAM assay. The group treated with Mat-TMZ showed a significant reduction (P=0.0262) in tumor area (-25890±9084 pixels^2^) compared to the control (1526±6752 pixels^2^) (Figure 7).

## Discussion

The large number of patents filed in recent years (17) demonstrates the extensive search for new compounds that are more effective in the treatment of glioblastoma. This study aimed to elucidate the mechanism of action of Mat, a compound isolated from *Miconia chamissois* Naudin, in combination with the chemotherapeutic of choice in clinical practice, TMZ, against a human glioblastoma cell line (U-251 MG) both *in vitro* and *in vivo*.

TMZ is a prodrug belonging to the triazene class that is able to cross the BBB and reach the tumor site in therapeutically relevant concentrations (18). Unlike other triazene compounds, TMZ does not require metabolic activation and is spontaneously converted to an active compound at blood pH (19). Although TMZ is used clinically to treat glioblastoma, the molecular mechanisms of action are not yet fully understood and are debated in the literature (20). Due to tumor heterogeneity, some tumor cells have mechanisms that can lead to resistance to this type of therapy. For example, mutations in *TP53* (a gene that encodes a protein involved in the induction of DNA repair systems) may prevent the death of tumor cells and the MGMT (O^6^-methylguanine methyltransferase) enzyme expression pattern is another factor in chemoresistance to TMZ (21). An effective therapeutic approach to avoid the development of resistance to TMZ is the use of different drug combinations in an attempt to resensitize the resistant tumor cells to treatment, by different mechanisms of action (22).

Several described flavonoids seem to induce apoptosis in cancer cells through intrinsic and extrinsic pathways (23). In glioblastoma, flavonoids also target other pathways, including activation of cell surface death receptors (24), such as kaempferol, which enhances TRAIL-mediated apoptosis through the extrinsic and intrinsic apoptotic pathways (25), and quercetin, which sensitizes glioma cells to death receptor-mediated apoptosis by suppressing the inhibitor of the apoptosis protein surviving (25). Although many studies have discussed the antitumor potential of flavonoids against glioblastoma, only one study, from our research group, demonstrated that the antitumor effects of Mat were due to the selective cytotoxicity of the compound and its potential to reduce important parameters such as migration, invasion, and clonogenicity, in addition to its ability to trigger the intrinsic apoptosis pathway (5). We also observed that this compound was able to reduce tumor growth and angiogenesis *in vivo* and demonstrated a synergistic cytotoxic effect when combined with TMZ (Mat-TMZ).

*In vitro* cytotoxicity tests are commonly used in early stages of the development of new drugs (26). We found that Mat-TMZ was highly cytotoxic and selective for tumor cells. This result was more significant compared to that obtained in another study using Mat (28 μg/mL) and TMZ (9.7 μg/mL) in isolation (5). It is interesting to note that the IC_50_ value of Mat alone was highly cytotoxic (9.56 μg/mL) against normal human astrocyte (NHA) cells (5), but when in combination with TMZ, it showed a considerable decrease (95%) in its toxicity. The sensitivity of tumor cells and lack of sensitivity of normal cells to antineoplastic treatments is a desirable feature for new therapies, since most treatments affect cells indiscriminately, leading to side effects that often limit their clinical use (3). TMZ is an example that, despite being used in glioblastoma therapy, has low selectivity for tumor cells (27), leading to several side effects such as nausea, vomiting, constipation, anorexia, alopecia, fatigue, convulsions, and neutropenia (28). We therefore highlight again the selectivity of Mat-TMZ against glioblastoma cells, since this is a desirable and rare ability in the field of new drug development.

Cell morphology changes were observed after treatment with Mat-TMZ, including chromatin aggregation, nuclear and cytoplasmic condensation, bubble formation, and changes in the permeability of the plasma membrane. Some of the observed changes, such as nucleus condensation and cell fragmentation, are typical characteristics of apoptosis (9). Thus, the morphological results obtained by the AO/PI test suggested the triggering of the apoptotic pathway in glioblastoma cells after treatment with Mat-TMZ. Several studies have demonstrated the activation of apoptosis by other flavonoids combined with TMZ, such as the combination of quercetin (30 μM) with TMZ (100 μM), in addition to significantly reducing cell viability (as demonstrated with Mat-TMZ in this study) through the inhibition of Hsp27 that sensitize these cells to apoptosis (29).

A study has shown that the combination of polyphenols and TMZ increased the antitumor activity with a lower concentration of TMZ, which increased apoptosis induction (30). Corroborating those studies, this study showed that Mat-TMZ may act in a similar way, inducing apoptosis of glioblastoma cells. Furthermore, a previous study by our research group (5) demonstrated that Mat alone led to an imbalance between pAKT (phosphorylated protein kinase B) and pERK (protein kinase B (PKB), and also increased capase-3/7 expression. Therefore, we believe that Mat-TMZ may act in a similar way to that observed in previous studies of other flavonoids, inducing apoptosis of glioblastoma cells.

Apoptosis is one of the most commonly studied forms of programmed cell death, which regulation involves two major signaling pathways: intrinsic and extrinsic (31). In the extrinsic pathway, apoptosis is initiated on the cell surface upon activation of death receptors. Oligomerization initiates the recruitment of adaptor proteins, such as TRADD (TNFR type 1-associated death domain), and initiator caspases, such as caspase-8 and/or caspase 10, forming the death-inducing signaling complex (DISC). This complex can subsequently activate the effector caspases such as caspase-3 (32). In addition, caspase-8 and caspase-10 can mediate proteolytic processing of BID into tBID favoring its translocation to mitochondrial membranes. This promotes cytochrome-c release into the cytosol (33).

Sequentially, cytochrome-c associated with APAF-1 and pro-caspase-9 forms the apoptosome complex leading to the activation of the intrinsic pathway of apoptosis (34). In this study, we aimed to investigate the role of Mat-TMZ upon tumor necrosis factor receptors (TNFR). There are two types of TNFRs, namely TNFR1 and TNFR2. Both receptors have different functions in cell signaling. The basic difference between TNFR1 and TNFR2 is the presence of the death domain (DD) linked to TNFR1. This association makes TNFR1 responsible for cell death activation, whereas TNFR2 is responsible for the induction of cell proliferation (35,36).

Docking energy results showed that both TMZ and Mat docked in the same cavity when analyzed with TNFR1 alone. When TNFR1 was combined with Mat, TMZ and Mat docked in other regions. These results suggested that these two molecules may act together increasing their potential. Another *in silico* study showed the affinity of some flavonoids to death receptors, including TNFR1 (37). Therefore, our data indicated that Mat-TMZ can initiate caspase cascade activation through the activation of the TNFR1 signaling pathway, leading to induction of both intrinsic and extrinsic apoptotic pathways.

Due to the success in producing a patient-derived tumor in the CAM model, Vu et al. (38) suggested this model as an alternative for screening anticancer drugs in a fast, efficient, and economical way. In this study, the Mat-TMZ combination was demonstrated to be effective in inhibiting tumor growth. There is evidence that TMZ alone has antiangiogenic properties (39 [Bibr B40]) and that Mat in isolation not only inhibits tumor growth and reduces tumor area r, but also decreases the blood vessel area and the number of junctions, characteristics of reduced angiogenesis (5). However, in this study, we did not observe these effects on angiogenesis (Figure S2).

In conclusion, this study demonstrated the synergistic anticancer activity of Mat, a compound isolated from *M. chamissois*, combined with the conventional drug TMZ (Mat-TMZ). This combination showed significant cytotoxic activity towards a human glioma cell line (U-251 MG), with high selectivity for tumor cells compared to normal cells (NHA). Mat-TMZ did not alter cellular adhesion in U-251 MG, possibly reducing the invasiveness of tumor cells. The study also demonstrated that the combination of these two compounds induced apoptosis, possibly through activation of TNFR1. Moreover, Mat-TMZ showed significant activity *in vivo*, reducing tumor growth after treatment. Eventually, these findings may lead to the development of new strategies to combat glioblastoma.

## Supplementary Material

Click to view [pdf].
